# Neurological and Medical Complications in Brain Tumor Patients. 2026 Update

**DOI:** 10.1007/s11910-026-01529-8

**Published:** 2026-11-09

**Authors:** Guneet Sarai, David Schiff

**Affiliations:** 1 Department of Neurosurgery, Division of Hematology Oncology, University of Louisville, Louisville, KY USA; 2 Neurological Surgery and Medicine, University of Virginia, 1215 Lee Street, Room 6225, Charlottesville, 22908-0432 VA USA

**Keywords:** Brain tumors, Complications, Seizures, Endocrine, Thromboembolism, Cognitive impairment

## Abstract

**Purpose of Review:**

Patients with brain tumors have a plethora of potential complications that arise from both the tumor and its therapeutic management. These complicate clinical management and produce morbidity and occasionally mortality. In this review we discuss the most common neurological and medical complications that include tumor-related epilepsy, vasogenic edema, infection, thromboembolism, endocrine abnormalities and cognitive impairment and its management. This review is an update to the 2020 article by Schiff and Alyahya.

**Recent Findings:**

IDH mutant gliomas cause neuronal hyperexcitability via D-2 -hydroxyglutarate production; use of IDH inhibitors may lower the rate of seizures in this patient population. Pneumocystis prophylaxis with antibiotics has been traditionally recommended with radiation and concurrent temozolomide chemotherapy but newer data suggest no difference in hospitalization or survival. Podoplanin produced by tumor cell releases into the circulation, often via extracellular vesicles, activates platelets and may contribute to venous thrombus formation. Direct oral Anticoagulants (DOACs) are the treatment of choice for venous thromboembolism, with high quality of evidence and a strong recommendation in cancer-associated thrombosis. Though brain tumor patients were underrepresented in clinical trials, retrospective studies support its safety in this population.

**Summary:**

Brain tumor patients are prone to complications arising from both the tumor and the treatment strategies. Management requires a multidisciplinary strategy with supportive medications including antiseizure medications, corticosteroids, anticoagulants for thromboembolism, and neurocognitive rehabilitation. Early recognition and timely intervention can reduce the morbidity associated with these complications leading to better patient outcomes.

## Introduction

A substantial proportion of patients with brain tumors develop short and long-term sequelae that can profoundly influence both survival and quality of life. The most common of these include epilepsy, stroke and other cerebrovascular events, cognitive impairment, vasogenic edema, radiation necrosis, fatigue, endocrine dysfunction, and infectious complications. In this review, we focus on pathophysiology and major recent advances in the understanding and management of these complications. This review is an update to the article published in 2020 [1].

## Tumor-Related Epilepsy

Tumor related epilepsy (TRE) is a major complication of brain tumors. Tumor growth and tumor directed treatments can affect seizure control. The incidence of epilepsy is higher in IDH-mutant tumors compared to IDH-wildtype tumors [2]. The putative mechanism is that D-2-hydroexyglutarate (D-2HG) produced due to IDH mutation causes neuronal hyperexcitability. The structural similarity of D-2HG to glutamine presumably activates N-methyl-D-aspartate leading to tumor growth. The putative mechanism causing increased neuronal hyperexcitability in tumor microenvironment is linked to excessive production of peritumoral glutamate. Glutamate causes hyperexcitability by stimulating the NMDA and the AMPA receptors of the neurons. While glutamate release causes seizures, this excitability also leads to a synaptic relationship between glia cell and neurons mediated through release of neuronal protein neuroligin-3 (NLGN-3) leading to further tumor growth [3, 4]. Inhibitors of the mutant IDH protein are increasing studied in their role not only to control tumor growth but also attain seizure control in low grade glioma patients. In the phase 3 INDIGO study of vorasidenib, among patients who had seizures while on study, those in the vorasidenib group had substantially lower rates of seizures than the placebo group [5].

Monotherapy antiseizure medication (ASM) should be started as soon as a patient with a brain tumor has a seizure. Levetiracetam remains the usual drug of choice as the initial ASM in this patient population. Selection of an ASM depends largely on the pharmacokinetic, pharmacodynamic, and adverse effects profile of each drug as discussed in the table, though can be prescriber specific. Table 1 summarizes mechanism of action, target dose, adverse effects and drug to drug interactions of ASMs.

**Table 1:** Commonly prescribed ASMs

ASM	Principal mechanism of action	Interactions	Target dose	Adverse effects	Idiosyncratic effects
Phenytoin	Sodium channel blocker	Hepatic enzyme inducer	200–400 mg/day guided by serum concentration	Somnolence, dizziness, ataxia, blurry vision, gum hyperplasia, irritability	SJS, lupus like reaction, hepatitis, blood dyscrasia
Carbamazepine	Sodium channel blocker	Hepatic enzyme inducer	Up to 800 mg/day	Nausea, headache, dizziness, fatigue, hyponatremia, weight gain, decreased bone density, mild leukopenia	SJS, TEN (more likely with HLA-B1502 allele), lupus like reaction, hepatotoxicity
Oxcarbazepine	Sodium channel blocker	Hepatic enzyme inducer	Up to 1200 mg/day	Similar to Carbamazepine (25% cross-reactivity). More likely to cause hyponatremia	Similar to Carbamazepine
Valproic acid	Multiple	Hepatic enzyme inhibitor	Up to 2000 mg/day	GI side-effects, tremors, alopecia, weight gain, PCOS, insulin resistance, hyperammonemia	Liver/pancreatic failure, thrombocytopenia, birth defects
Levetiracetam	SV2A modulation	n/a	Up to 4000 mg/day	Somnolence, dizziness, fatigue, irritability, depression	None established
Brivaracetam	SV2A modulation	More interactions than levetiracetam	Up to 200 mg/day	Somnolence, dizziness, fatigue, irritability	None established
Lamotrigine	Sodium channel blocker	Decrease OCP efficacy	4.5–7.5 mg/kg/day guided by serum concentration	Dizziness, blurry vision, ataxia, headaches, insomnia	SJS, TEN, DRESS
Topiramate	Multiple	Decrease OCP efficacy	Up to 400 mg/day	Somnolence, dizziness, mental slowing, memory disturbance, weight loss, kidney stones	Hepatic failure, glaucoma
Lacosamide	Sodium channel blocker (enhances slow inactivation)	n/a	Up to 400–600 mg/day	Somnolence, dizziness, diplopia, headaches, tremors	None established
Zonisamide	Multiple	n/a	Up to 600 mg/day	Somnolence, ataxia, dizziness, decreased appetite, paresthesia, weight loss, kidney stones, apathy	Rash
Perampanel	Noncompetitive AMPA glutamate receptor antagonist	Decrease OCP efficacy	8–12 mg/day	Dizziness, somnolence, headache, fatigue, ataxia, blurred vision, aggression and hostility	DRESS
Clobazam	Potentiate GABAergic neurotransmission	n/a	Up to 40 mg/day	Drowsiness, dizziness, withdrawal seizures	None established
Cenobamate	Dual sodium channel blocker (persistent inactivation)/GABAergic	n/a	up to 400 mg/day	Dizziness, ataxia, double vision, headaches	DRESS

Adapted from Schiff and Alyahya (2020) [reference # 1]

First generation ASMs (carbamazepine, phenytoin, phenobarbital, and primidone) are routinely avoided in TRE due to their cytochrome P450 enzyme inducing properties and drug-drug interactions. Use of valproic acid in TRE has declined over the years but is still used as second- or third-line treatment for uncontrolled seizures [4]. Second generation ASMs include levetiracetam, lamotrigine, topiramate, oxcarbazepine and gabapentin. The popularity of levetiracetam is largely driven by its favorable pharmacokinetic profile and lack of drug-drug interactions and monitoring requirements. Lamotrigine is well tolerated medication and often prescribed to women of childbearing age with seizures due to its lack of discernible risk of contributing to fetal birth defects. Lamotrigine needs titration and often needs bridging with another ASM [4].

Topiramate at seizure prevention doses can cause cognitive slowing and weight loss. Zonisamide is well tolerated by most patients though weight loss is commonly reported and should be avoided in patients with nephrolithiasis. Oxcarbazepine, approved for focal seizures in adults and children, is derivative of carbamazepine with favorable side effect profile. There are limited data on gabapentin and pregabalin in seizure control in TRE [4].

The third generation ASM lacosamide is well tolerated and used as monotherapy for control of TRE. It is effective both as monotherapy and as add on therapy in TRE. Eslicarbazepine is related to carbamazepine and oxcarbazepine with once-daily dosing and has favorable side effects but studied as add-on therapy only in TRE. Brivaracetam is an analog of levetiracetam that is used sometimes when levetiracetam produces mood side effects. Clobazam and perampanel are the other third generation ASMs with smaller studies in TRE. Finally, cenobamate has some use in refractory epilepsy but has not been studied in TRE [4].

Previous hopes that the histone deacetylase activity of valproic acid might have antitumoral effects in glioblastoma based upon a retrospective EORTC 26,981–2298 study and a small phase 2 trial have not been borne out [6, 7]. Levetiracetam and the AMPA receptor inhibitor perampanel have both been studied for antitumor effects in glioma; however, despite early studies indicating benefit, subsequent studies failed to confirm therapeutic benefit in glioma patients [8].

Prophylactic ASMs are not routinely recommended in brain tumor patients. ASMs continue to be used perioperatively for tumor craniotomies though some retrospective studies have debated its benefit [9, 10]. Withdrawal of ASM can be attempted on patients who had gross total resection of tumor and no evidence of tumor growth after finishing tumor directed treatments [11]. Steroids are heavily used in the brain tumor populations but have no role per se in treatment of seizures.

## Vasogenic Edema

Management of vasogenic edema remains a major challenge in the clinical practice of neuro-oncology. Its pathophysiology remains complex with multiple factors and potential therapeutic targets, including vascular endothelial growth factor (VEGF), nitric oxide (NO), arachnoid acid metabolites, and aquaporins (AQP4) [12].

Dexamethasone’s biological half-life lasts up to 54 h, and thus outpatient administration twice daily is feasible [13]. Among its numerous potential side effects are insomnia, weight gain, diabetes mellitus, myopathy, osteoporosis, and peptic ulceration. Dexamethasone use is also associated with shortened survival in glioblastoma patients [4, 14]. Moreover, a large retrospective study of perioperative dexamethasone was associated with decreased overall survival in brain metastases patients undergoing resection [15]. At each encounter the clinician should consider lowering the dose of steroids though the reduction should be individualized according to patient response.

Bevacizumab is used for management of cerebral vasogenic edema of different etiologies and has gained popularity as a steroid-sparing agent [16]. Steroids can also be problematic in brain metastases from underlying malignancies treated with immunotherapy as it may reduce efficacy of checkpoint inhibitors [17], though the importance of this observation is debated [18]; the use of bevacizumab as a steroid sparing agent can be considered in such cases [19]. Recently receptor for advanced glycation end products (RAGE) inhibitors have shown to decrease cerebral edema in mouse models without side effects of steroids [20]. 

## Infectious Complications

Infectious complications are not uncommon in brain tumor patients. Lymphopenia from corticosteroids and chemotherapy like temozolomide can be profound and long lasting, determining prognosis in patients [21, 22]. Pneumocystis pneumonia is associated with lymphopenia, particularly with lymphocyte counts < 500 per microliter. Antibiotic prophylaxis, most commonly with sulfamethoxazole/trimethoprim, has been traditionally recommended with radiation and concomitant temozolomide. A retrospective study with over 3000 patients showed that the number needed to treat was 288, with no difference in hospitalization or survival with or without the use of prophylaxis, suggesting reconsideration of prophylaxis [23]. Other infections related to immunosuppression are occasionally seen and include herpes reactivation [24], CMV-related infections [25], and Aspergillosis [26]. 

## Vascular Complications

Glioma patients carry one of the highest risks of venous thromboembolism (VTE) among cancer patients, with rates up to 25–39% [27–29]. In a large prospective multicenter study of newly diagnosed glioblastoma, symptomatic VTE occurred in 24% of patients, particularly in the first 6 months after diagnosis [30]. Even patients with lower-grade gliomas are not immune to thrombotic events, though patients with IDH wildtype tumors are at greater risk of VTE than those with IDH mutation [31]. Several VTE risk factors have been identified in gliomas include increasing age, prior history of VTE, limb paresis, poor performance status, glioma-specific factors (IDH wildtype, tumor > 5 cm, supratentorial location) and treatment related (subtotal resection, chemotherapy, steroid use, bevacizumab use) [30]. 

The pathophysiology of venous thromboembolism (VTE) in glioma patients relates to tumor-driven hypercoagulability. Glioblastomas overexpress procoagulant proteins such as tissue factor (TF) and podoplanin (PDPN) [32]. A plausible mechanism extrapolated from murine studies is that PDPN release into the circulation, often via extracellular vesicles, activates platelets through interaction with the CLEC-2 receptor leading to thrombus formation. IDH-mutant gliomas exhibit reduced expression of these procoagulant factors due to epigenetic modifications, DNA histone methylation by 2HG production, resulting in a comparatively lower risk of thrombosis [33].

Any pain, swelling or cramping in lower extremities, dyspnea and fatigue warrants the need to rule out VTE by lower extremity venous doppler and potentially CT pulmonary angiogram [30]. Pulmonary embolism (PE), a life-threatening condition of VTE needs to be diagnosed in a timely manner. Tools like the Wells score and Khorana scale have been used in clinical practice but have failed validation in glioblastoma patients [34]. A predictive time-to-event tool for VTE in glioma patients that includes 10 variables has been proposed by Burdett and Horbinski but requires independent validation [35].

VTE can be challenging to treat and is associated with worse survival in glioblastoma patients [29]. Direct oral anticoagulants (DOACS) are now the treatment of choice as per the NCCN guidelines for treatment of cancer associated thrombosis (CAT) in patients without gastric or gastroesophageal lesions. Apixaban and rivaroxaban were added as options for extended pharmacologic thromboprophylaxis after cancer surgery [36, 37]. 

The major complication of anticoagulation use in brain tumor patients is intracranial hemorrhage (ICH). Several retrospective studies have examined the safety of low molecular weight heparin (LMWH) and DOACS in VTE in brain tumor patients. A few studies found that LMWH for VTE in glioblastoma patients increased risk of ICH compared to glioblastoma patients who are not on anticoagulation [38, 39]. Other retrospective studies failed to show increased risk of ICH with LMWH in glioma patients compared to glioma patients not on anticoagulation [40, 41]. DOACs showed a satisfactory safety profile in a small retrospective study of 42 patients with VTE and glioblastoma [42]. Additional findings from a retrospective study of 121 patients demonstrated lower incidence of ICH in DOACS cohort [43]. Most of the prospective phase 3 trials to establish safety of DOACS in CAT excluded or underrepresented brain tumor patients. Based on the retrospective data to date, use of DOACS to treat VTE In brain tumor patients is likely safe [44].

The widespread use of the VEGF antagonist bevacizumab has raised the concern that this drug might increase VTE risk. However, a metanalysis found no significant difference in risk of DVT (deep vein thrombosis) and PE in patients treated with bevacizumab compared to control groups though there was a trend (*p* = 0.07) towards increased risk of PE only [45]. Bevacizumab increases the risk of cerebral infarcts as well as hemorrhages in cancer patients generally independent of other risk factors [46]. A recent small retrospective study that investigated the risk of ischemic strokes and hemorrhages in patient treated for recurrent glioblastoma with or without bevacizumab failed to demonstrate an association between bevacizumab and CNS vascular events [47]. However, in the AVAGlio study for newly diagnosed glioblastoma, the risk of serious intracranial hemorrhage was 2% in the bevacizumab arm and 0.9% in the placebo arm [48]. Posterior reversible encephalopathy syndrome (PRES) has been reported in patients with primary or metastatic brain tumors or metastases receiving chemotherapy, most commonly VEGF inhibitors [49–51].

## Endocrine and Fertility Complications

Endocrinopathies and hypothalamic-pituitary axis dysfunction are common in brain tumor patients with an incidence that may exceed 30% if the radiation field involves the hypothalamus and pituitary gland. Dysfunction typically starts within a few years of radiation and is radiation dose dependent [52], manifesting as decreased production of growth hormone, thyroid stimulating hormone, adrenocorticotropic hormones, and gonadotropins. According to a retrospective study, cranial irradiation of 18 Gy or more in children was associated with obesity and 30 Gy or more was associated with adrenal insufficiency compared to general population [53].

Patients with brain tumors are at risk of infertility due to direct or secondary effects of radiation or chemotherapy. Nonetheless, a recent small retrospective study of young adult patients with brain tumors, mainly gliomas, found that only 24% of eligible patients were referred to fertility specialists [54]. Fertility risk and preservation counseling should be discussed with all patients of reproductive age prior to treatment and available strategies should be incorporated to discuss fertility preservation at the time of clinic visits.

## Cognitive and Behavioral complications

Patients with cancer are living longer due to advances in therapeutic strategies but with increased incidence of neurocognitive decline secondary to neoplasm or treatment. Multiple studies have shown pretreatment cognitive impairment is present in patients with non-CNS malignancies with approximately 30% of patients having detectable impairment before treatment, 75% during and 35% over the years after completion of treatment [55–59]. 

Peripheral cancer related inflammation impairs cognitive function through multiple interconnected putative mechanisms that includes cytokine (IL-1B, IL-2, IL-6, TNF-alpha, IFN) mediated disruptions of synaptic function, tumor derived extracellular vesicles carrying inflammatory microRNA across blood brain barrier and neutrophilic infiltration of velum interpositum [60]. Genetic factors such as the APOE ɛ4 allele have been studied and may predispose to impairment though this is debated [61]. Serum ApoJ, ApoE, or ApoA protein and higher amyloid beta (Aβ 1–42) levels have also been studied in cognitive decline [62].

Chemotherapy plays a contributory role in cognitive impairment, and numerous studies have reported reduction in gray matter and less connectivity and activation of neurons following chemotherapy [63, 64]. Memory, processing speed, attention and executive functions are the domains that are most affected by chemotherapy [65] Cognitive decline is accelerated in older cancer survivors (> 65 years of age) [66]. Whole brain radiation therapy (WBRT) causes more cognitive impairment than focal radiation [66]. Immunotherapy and recently emerging treatments such as chimeric antigen receptor T (CAR T) cell therapy and bispecific T cell engager therapy (BiTE) often leads to neurocognitive complications in patients as part of (ICANS) encephalopathy with confusion, seizures, behavior, and language problems [67].

Efforts to mitigate the deleterious effects of treatment in patients with brain tumors are actively being studied. The phase 2 study RTOG 0933 demonstrated that hippocampal avoidance during WBRT (HA-WBRT) produced a significantly lower mean relative decline of cognition than WBRT and is the preferable method of WBRT delivery [68]. These findings were confirmed in a phase 3 trial with prophylactic cranial irradiation in small cell lung cancer patients of WBRT with or without hippocampal avoidance. Delayed free recall decline was lower in the hippocampal avoidance arm at 3, 6, 12, and 24 months [69]. Additional supporting evidence came from the phase 3 trial NRG CC001 randomizing patients with brain metastases to HA-WBRT plus memantine versus WBRT plus memantine. Although primary endpoints were not achieved, the HA-WBRT arm experienced better preservation of cognitive function and patient-reported symptoms [70]. A subsequent exploratory analysis of NRG CC001 found differential neuroprotective response to HA-WBRT with patients surviving more than 4 months and or lung histology having lower neurocognitive function failure. Decreasing the volume of brain treated with radiation represents another technique to mitigate cognitive damage. Stereotactic radiosurgery to the surgical cavity post resection for brain metastases appears safe with no significant cognitive decline compared to WBRT [71]. A recent systematic review reiterated that HA-WBRT or SRS decreased cognitive decline and improved quality of life without increased toxicity in a few of the evaluated trials in the study [72].

The NMDA receptor antagonist and cholinesterase inhibitor donepezil are both approved for Alzheimer’s disease. These drugs have also been studied in cognitive decline from WBRT in brain tumor patients. HA-WBRT plus memantine was associated with sustained preservation of cognitive function even at 12 months and fewer symptoms over time though with no difference in survival data [73] Donepezil had improvement in some cognitive domains in smaller phase 2 trials but has not significantly shown benefit in larger phase 3 trial [74, 75].

Other non-pharmacological treatments such as cognitive behavioral therapy (CBT) and neurocognitive rehabilitation may be helpful in selected patients [76]. There are ongoing trials studying different agents and techniques to mitigate neurocognitive impairments in patients undergoing radiation for brain metastases treatment. A randomized, controlled trial evaluated the effects of a multifaceted cognitive rehabilitation program (CRP) on cognitive functioning and selected quality-of-life domains in patients with gliomas. At the 6-month follow-up, the CRP group performed significantly better than the control group on neuropsychological tests of attention and verbal memory and reported less mental fatigue [77]. Cognitive behavior therapy has also shown reduce to fatigue in glioma patients and could be a promising strategy for cancer patients.

## Conclusion

Brain tumors are associated with a wide range of medical and neurological complications arising from both the tumor and the therapies used to treat it. Prompt recognition and timely intervention are critical in managing the complications to mitigate the neurological morbidity and minimize treatment interruptions. Continued research into understanding the mechanism of tumor related complications and targeted treatment options is needed to improve quality of life and long-term outcomes in brain tumor patients.

## Key References

- Cloughesy TF, van den Bent MJ, Touat M, Blumenthal DT, Peters KB, Ellingson BM, et al; INDIGO trial investigators. Vorasidenib in IDH1-mutant or IDH2-mutant low-grade glioma (INDIGO): secondary and exploratory endpoints from a randomised, double-blind, placebo-controlled, phase 3 trial. Lancet Oncol. 2025 Dec; 26:1665-75. https://doi.org/10.1016/S1470-2045(25)00472-3
  -  ○IDH mutation inhibitors can decrease incidence of seizures in patients with IDH mutant tumors.
- Tawil N, Bassawon R, Meehan B, Nehme A, Montermini L, Gayden T, et al. Glioblastoma cell populations with distinct oncogenic programs release podoplanin as procoagulant extracellular vesicles. Blood Adv. 2021 Mar 23;5(6):1682-94. https://doi.org/10.1182/bloodadvances.2020002998
  -  ○Plausible mechanism of venous thromboembolism in cancer is that PDPN release into the circulation, often via extracellular vesicles, activates platelets through interaction with the CLEC-2 receptor leading to thrombus formation.
- Gondi V, Deshmukh S, Brown PD, Wefel JS, Armstrong TS, Tome WA, et al. Sustained preservation of cognition and prevention of patient-reported symptoms with hippocampal avoidance during whole-brain radiation therapy for brain metastases: final results of NRG Oncology CC001. Int J Radiat Oncol Biol Phys. 2023;117(3):571-80. https://doi.org/10.1016/j.ijrobp.2023.04.030
  -  ○HA-WBRT benefits patients surviving more than 4 months and/or with lung histology having lower neurocognitive function failure

## Data Availability

No datasets were generated or analysed during the current study.
